# Psychometric Properties of the Functional Social Support Domain of Perinatal Infant Care Social Support

**DOI:** 10.17533/udea.iee.v38n2e04

**Published:** 2020-07-10

**Authors:** Carolina Vargas-Porras, Zayne Milena Roa-Díaz, Carme Ferré-Grau, María Inmaculada De Molina-Fernández

**Affiliations:** 1 Nurse, Masters, Ph.D Candidate, Universitat Rovira i Virgili, Departament d’Infermeria, Tarragona, Spain. Full Professor, Universidad Industrial de Santander, Bucaramanga, Colombia. Email: carvarpo@uis.edu.co Universitat Rovira i Virgilu Universitat Rovira i Virgili Spain carvarpo@uis.edu.co; 2 Nurse, Masters, Ph.D student. University of Bern, Bern, Switzerland. Email: zaynemilena6@gmail.com University of Bern University of Bern Switzerland zaynemilena6@gmail.com; 3 Nurse, Psychologist, Ph.D. Full Professor, Universitat Rovira i Virgili, Tarragona, Spain. Email: carme.ferre@urv.cat Universitat Rovira i Virgilu Universitat Rovira i Virgili Spain carme.ferre@urv.cat; 4 Nurse, Matron, Masters, Ph.D. Professor, Universitat Rovira i Virgili, Tarragona, Spain. Email: inmaculada.demolina@urv.cat Universitat Rovira i Virgilu Universitat Rovira i Virgili Spain inmaculada.demolina@urv.cat

**Keywords:** validation studies: psychometrics, reproducibility of the results, translating, mothers, social support., estudios de validación: psicometría, reproducibilidad de los resultados, traducción, madres, apoyo social., validation studies, psychometrics, reproducibility of results, translating, mothers, social support.

## Abstract

**Objective.:**

To determine the face, content, construct validity, and reliability of the functional social support domain of Perinatal Infant Care Social Support (PICSS) translated into Spanish and adapted for first-time mothers of term babies.

**Methods.:**

Validation study of the functional social support domain of PICSS, which has 22 items with response options from 1 to 4; higher scores indicate greater social support. A translation, back-translation, and cultural adaptation process took place along with an expert review to evaluate face and content validity. In total, 210 mothers participated to establish construct validity and the reliability of the domain. The content validity index and factor analysis were used to identify the structure of the domain. Reliability was estimated using Cronbach’s alpha coefficient.

**Results.:**

Linguistic and cultural adaptations were performed, along with validation and reliability. Face validity for mothers was the following: high comprehension (94%); and for experts: high comprehension (95.83%), high clarity (96.53%), and high precision (92.82%). In relevance and pertinence, the content validity index was high (0.97). Construct validation identified two factors that explained 76% of the variance of the domain evaluated: factor 1 “Supporting presence -emotional and appraisal support” (13 items, 39%) and factor 2 “Practical support -informational and instrumental support-” (9 items, 37%). Cronbach’s alpha value was 0.97.

**Conclusion.:**

Given the robust psychometric properties of the Spanish version of the functional social support domain of PICSS, this may be used to identify the functional social support in the mothers.

## Resumen

**Objetivo.:**

Determinar la validez facial, de contenido, de constructo y confiabilidad del dominio de apoyo social funcional del *Perinatal Infant Care Social Support* (PICSS) traducido al español y adaptado para madres primerizas de bebés a término.

**Métodos.:**

Estudio de validación del dominio de apoyo social funcional del PICSS, el cual tiene 22 ítems con opciones de respuesta de 1 a 4, cuanto más alto sea el puntaje es mayor el apoyo social. Se llevó a cabo un proceso de traducción, retrotraducción y adaptación cultural y una revisión por expertos para evaluar la validez facial y de contenido. Un total de 210 madres participaron para establecer la validez de constructo y la confiabilidad del dominio. Se utilizó el índice de validez de contenido y el análisis de factores para identificar la estructura del dominio. La confiabilidad se estimó mediante el coeficiente alfa de Cronbach.

**Resultados.:**

Se realizaron adaptaciones lingüísticas y culturales, validación y confiabilidad. La validez facial para las madres fue la siguiente: alta comprensión (94%); y para expertos: alta comprensión (95.83%), alta claridad (96.53%) y alta precisión (92.82%). En relevancia y pertinencia el índice de validez de contenido fue alto (0.97). La validación de constructo identificó dos factores que explicaron el 76% de la varianza del dominio evaluado: factor 1 “Presencia de apoyo -apoyo emocional y de valoración” (13 ítems, 39%) y factor 2 “Apoyo práctico -apoyo informativo e instrumental-” (9 ítems, 37%). El valor alfa Cronbach fue 0.97.

**Conclusión.:**

Dadas las robustas propiedades psicométricas de la versión en español del dominio de apoyo social funcional del PICSS este puede usarse para identificar en las madres el apoyo social funcional.

## Resumo

**Objetivo.:**

Determinar a validez facial, do conteúdo, de construto e confiabilidade do domínio de apoio social funcional do Perinatal Infant Care Social Support (PICSS) traduzido ao espanhol e adaptado para futuras mães de bebés a término.

**Métodos.:**

Estudo de validação do domínio de apoio social funcional de PICSS, o qual tem 22 itens com opções de resposta de 1 a 4, quanto mais alto seja a pontuação é maior o apoio social. Se levou a cabo um processo de tradução, retro-tradução e adaptação cultural de acordo e uma revisão por especialistas para avaliar a validez facial e de conteúdo. Um total de 210 mães participaram para estabelecer a validez de construto e a confiabilidade do domínio. Se utilizou o índice de validez de conteúdo e a análise de fatores para identificar a estrutura do domínio. A confiabilidade se estimou mediante o coeficiente alfa de Cronbach.

**Resultados.:**

Se realizaram adaptações linguísticas e culturais, validação e confiabilidade. A validez facial para as mães foi a seguinte: alta compreensão (94%); e para especialistas: alta compreensão (95.83%), alta claridade (96.53%) e alta precisão (92.82%). Em relevância e pertinência o índice de validez de conteúdo foi alto (0.97). A validação de construto identificou dois fatores que explicaram 76% da variável do domínio avaliado: fator 1 “Presença de apoio -apoio emocional e de valorização” (13 itens, 39%) e fator 2 “Apoio prático -apoio informativo e instrumental-” (9 itens, 37%). O valor alfa Cronbach foi de 0.97.

**Conclusão.:**

Dadas as robustas propriedades psicométricas da versão em espanhol do domínio de apoio social funcional de PICSS este pode usar-se para identificar nas mães o apoio social funcional.

## Introduction

When a woman becomes a mother, she faces big challenges regarding the new role with extensive physical, psychological, and social work(1) and, consequently, she must readjust her daily routine and priorities to respond to her care and that of her child.(2) For a first-time mother, this transition is more difficult because of not having prior experience.(3) 

Research findings indicate that first-time mothers require social support during the transition to maternity(4-6) Social support may be functional and structural. Functional support refers to exchange activities in a relationship and this support is divided - in turn - into informational (information exchanged between individuals or a group, which has a positive result for the recipient), instrumental (transactions in which direct aid or assistance is offered), emotional (emotional concern for the recipient), and of appraisal (statements or expressions of agreement or correction of some action or point of view). Structural support are the support sources or networks, which can be: formal, like the support offered by the health staff, or informal, especially from their partner or mother.(7) 

Social support reduces the tension generated by the new maternal role that favors the affective bond,(8) it is associated with greater confidence of the mother(9) and diminishes the risk of postpartum depression.(10) Bearing in mind the positive impact of social support on the maternal and child health, it is a challenge for nursing to design interventions that favor the different types of social support in first-time mothers and evaluate their effectiveness, which require valid and reliable scales. The literature reviewed by the authors showed no valid and reliable instrument in Spanish that measured specifically the different types of social support in first-time mothers with term babies, within the context of infant care during the postpartum period. 

Perinatal Infant Care Social Support (PICSS)(10) is an instrument in English, designed to measure social support in first-time mothers within the context of infant care practices, supported by the theory of social support. The PICSS is comprised by two domains, one to identify structural support, and another to identify functional support, having adequate validity and reliability;(11) however, the PICSS has not been translated, adapted, or validated into Spanish. Considering that the structural domain of the PICSS is not susceptible to psychometric tests(11) and that the functional domain of the PICSS measures different types of support, the aim of this study was to determine the face, content, and construct validity, as well as the reliability of the functional social support domain of the PICSS translated into Spanish and adapted for first-time mothers of term babies.

## Methods

Validation study conducted in Colombia during 2018. The participants were contacted in the puerperium service and in outpatient consultation at the San Luis Materno Infantil Clinic in Bucaramanga, an institution attending women from different cities. The inclusion criteria were: first-time mothers with only child, to term, healthy and who were in the first six months postpartum. Mothers with morbidities were excluded.

Domain of functional social support of the PICSS. This was created and validated by Nurse Patricia Leahy-Warren of the University College Cork, Ireland. It is comprised by 22 items distributed into four dimensions (informational support, instrumental support, emotional support, and support of appraisal) evaluated using a four-point Likert-type scale (totally disagree = 1; disagree = 2; agree = 3; totally agree = 4). The minimum score is 22 and the maximum is 88, with higher scores meaning greater social support.(10,11)

Translation, back-translation, and cultural adaptation. The study followed the guidelines by Muñiz *et al*(12) The translation of the original version of the Functional Social Support domain from English to Spanish was made independently by two bilingual nurses and an official translator. Upon obtaining the three translations, the review committee (official translator, philologist, and three expert nurses in maternal-infant health) compared the translations and in consensus originated the initial version, according to the agreement between the original semantics of the questionnaire and the comprehension of each of the items and according to the context of the study population. This Spanish version of the domain was back-translated by another official translator and by two bilingual nurses, who did not know the version in English. With the three back-translations, the review committee reached consensus on the initial version. It was delivered to the author of the PICSS, the initial version into Spanish, together with the initial version of the back-translation for its approval. Thereafter, the adjustments requested were made and the review committee in consensus originated the second version of the domain, which was approved by the author of the PICSS and, then, tested in a pilot study with 10 first-time mothers to obtain the definitive version of the functional social support domain of the PICSS, translated into Spanish and with cultural adaptation. 

Experts. To select the experts, the study considered the classification criteria by Fehring,(13) according to which a minimum score of 5 is needed from the total of 14 to be considered an expert, thus: PhD (4 points), Masters (3 points), Specialization (2 points), article published on maternal-infant health (1 point), teaching experience of at least one year in maternal-infant health (1 point), professional experience of at least one year in maternal-infant health (2 points), research in the area of maternal-infant health (1 point). This information was evaluated through the *curriculum vitae* available on the webpage of Colombia’s Ministry of Science, Technology, and Innovation. Upon selecting the experts, they were sent an e-mail letter inviting them to participate. The first validation round had participation from 27 experts (7 with PhD, 16 with Masters, and 4 with Specialization) from 17 universities corresponding to 11 capital cities in Colombia; the second round had participation from 4 experts (1 with PhD, 2 with Masters, and 1 with Specialization) different from those in the first round, from four universities corresponding to four capital cities. The average of years of teaching experience in the area of maternal-infant health was 12 years (range: 4 - 29), of professional experience in the area of maternal-infant health was 16.77 years (range: 8 - 37), of number of articles published in the area of maternal-infant health was 4.59 (range: 3 - 22), of number of investigations conducted in the area of maternal-infant health was 5.03 (range: 3 - 20).

Face validity. This validity saw participation from another 10 first-time mothers who were between the first and sixth month postpartum, belonging to different socioeconomic and educational levels. The mothers evaluated the criterion of comprehension, with the score: 1 = I don’t understand it, 2 = I understand it poorly, and 3 = I understand it. And the experts, in addition to this criterion, evaluated the criterion of clarity, with the score: 1 = it is not clear, 2 = it is not very clear, and 3 = it is clear. Lastly, they also evaluated the criterion of precision, with the score: 1 = it is not precise, 2 = it is not very precise, and 3 = it is precise(14) The degree of comprehension, clarity, and precision of the items was determined through percentages: High = equal to above 85%, Median = 80% - 84.9%, and Low = equal to or below 79%.

Content validity. The experts evaluated the criterion of pertinence, with the score: 1 = not pertinent, 2 = poorly pertinent, 3 = pertinent, 4 = very pertinent. And the criterion of relevance, with the score: 1 = not relevant, 2 = poorly relevant, 3 = relevant, 4 = very relevant(15) The content validity index (CVI)(16) was calculated for each expert with the following formula: number of items with a score entre 3-4 divided between the total number of items, followed by the estimation of the general content validity index, using the formula: sum of the CVI calculated for each expert divided between the total number of experts. The CVI for each item was determined to evaluate their pertinence and relevance. The calculation was made by using the formula: number of experts agreeing on the relevance value or the pertinence value of each item divided between the numbers of experts. A quantitative analysis was performed of the content validity by bearing in mind that scores equal to or above 0.80 have high content validity.(16) 

Thereafter, a qualitative analysis was made of the observations given in the first round of experts to each of the items in the following manner: in the second round, a group of experts from the area of maternal-infant health different from that participating previously, to control information selection bias (that is, they would be inclined to prioritize their own observations), reviewed each of the observations and through consensus agreed. Required adjustments were made in the different items. To make modifications in an item, agreement consensus was needed from over 50% of the experts.

For the construct validity and reliability, sample size was determined according to the criterion of 10 participants by the number of items in the scale(17) The final number of participants analyzed in this study was 210. Mean age was 24.39 years (SD±5.66). The participants belonged to socioeconomic levels: 1 and 2 (47.62%), 3 and 4 (49.05%), and 5 and 6 (3.33%); 39.05% were housewives; 25.71% were employed and 35.24% performed other activities; 35.24% had university formation; 33.33% high school formation and 28.57% technical or technological formation; only 2.86% had only primary education; most were in common-law relationships or were married (86.19%). The type of delivery was vaginal (51.43%) and the rest via cesarean (48.57%). On the moment of collecting the information, 87.61% of the mothers had less than a month of postpartum. 

Construct validity. The factor analysis began with the exploration of the total correlations of the items through Pearson’s correlation coefficient, followed by the application of Bartlett’s sphericity test and calculation of sample adequacy through the Kaiser-Meyer-Olkin (KMO) statistic, which considered acceptable a coefficient > 0.65.(18) Factor extraction was conducted by considering a minimum value of 0.3 in the correlation coefficients of the factors and eigenvalues >1 to be considered important; also, an explained variance >60% was expected, varimax orthogonal rotation was used. Analyses were conducted in Stata v12.0. 

Reliability. Cronbach’s alpha coefficient was used to calculate the estimations of internal consistency in the total sample and in each of the dimensions of the functional social support domain of PICSS. A coefficient of 1.00 indicates a perfect reliability and a coefficient of 0.00 indicates reliability does not exist.(19)

Ethical aspects. The study adhered to Resolution 008430 of 1993 by the Colombian Ministry of Health, which establishes the standards for health research. Furthermore, the study kept in mind the international ethics guidelines for research on human beings as mandated by the Helsinki Declaration. All the participants submitted a written informed consent. This research was approved by Universitat Rovira i Virgili in Spain and by the Hospital Bioethics Committee at the San Luis Materno Infantil Clinic in Bucaramanga, Colombia.

## Results

Face validity and content validity. The face validity score by the mothers was: high comprehension (94%). The face validity score by the experts was: high comprehension (95.83%), high clarity (96.53%) and high precision (92.82%). In relevance, the content validity index was 0.97 and in pertinence, the content validity index was also 0.97. Adjustments were made of the items by consensus agreement. 

Construct validity. The model showed simple adequacy (KMO = 0.94, Bartlett’s test *p* <0.001), thus, the factor analysis was performed. Pearson's pairwise correlations of all domain items had values between 0.35 and 0.94. Two factors were determined with eigenvalues >1: the first with 14.47 and the second with 2.22, which after the varimax orthogonal rotation explained 39% and 37% of the variance, respectively, for an accumulated 76% explained variance. Bearing in mind the items that compose each of the factors in the rotated matrix, factor 1 is named “Supporting presence (emotional and appraisal support)” (13 items) and factor 2 is named “Practical support (informational and instrumental support)” (9 items). In both factors the factor loads were above 0.62. Communality values for all the items were in the range of 0.36 and 0.88 (Table 1).

**Table 1:** Higher score on factor loads with varimax orthogonal rotation

Items	Factor 1	Factor 2	Communalities
V01- I can get information on how to feed the baby		0.88	0.87
V02- I can get information on how to change the nappies / dress the baby		0.89	0.88
V03- I can get information on how to console the baby/make the baby comfortable		0.87	0.88
V04- I can get information on how to bathe the baby		0.86	0.84
V05- I can get information on taking care of my body after child birth		0.64	0.73
V06- I can learn from other mothers’ experiences	0.70		0.76
V07- I can get information regarding baby care	0.71		0.78
V08- I can get hands on help with my baby to feed the baby		0.75	0.65
V09- I can get hands on help with my baby to change the nappies / dress the baby		0.85	0.84
V10- I can get hands on help with my baby to console the baby / make the baby comfortable		0.85	0.88
V11- I can get hands on help with my baby to bathe the baby		0.81	0.75
V12- I have someone to help me with routine housework	0.62		0.55
V13- I won't be on my own taking care of my baby	0.69		0.66
V14- I have time for myself	0.55		0.36
V15- I have people I can count on when things go wrong	0.84		0.85
V16- I have someone who takes care of and concerns about me	0.84		0.72
V17- I have someone to talk to about how I am feeling	0.86		0.81
V18- If I need advice there is someone who will assist me to work out a plan for dealing with the situation	0.82		0.81
V19- I have people I can talk to and share my experiences with	0.81		0.80
V20- I have people who will show me appreciation for the care I give to my baby	0.84		0.86
V21- People close to me understand that it is okay for me to need help	0.80		0.80
V22- I can get positive feedback from health care professionals about my ability to care for my baby	0.72		0.61

Reliability. Cronbach’s alpha for all the functional social support domain was 0.97 (95%CI = 0.97-0.98). By dimension, factor 1 had a slightly inferior coefficient (0.96, 95%CI = 95.42-96.93) with respect to factor 2 (0.97, 95%CI = 95.80-97.80). Values per each item are shown in Table 2. 

**Table 2:** Varimax orthogonal rotated factors, item-test correlation and Cronbach’s alpha

Items in Spanish	Items in English	Item-test correlation	Total alpha if item is eliminated
Factor 1. Presencia de apoyo (apoyo emocional y de valoración)	Factor 1. Supporting Presence (emotional and appraisal support)		
V06-Puedo aprender de las experiencias de otras madres	I can learn from other mothers’ experiences	0.86	0.96
V07-Puedo obtener información sobre el cuidado del bebé	I can get information regarding baby care	0.87	0.96
V12-Tengo a alguien que me ayuda con las tareas domésticas de rutina	I have someone to help me with routine housework	0.76	0.96
V13-No estaré sola cuidando a mi bebé	I won't be on my own taking care of my baby	0.83	0.96
V14-Tengo tiempo para mí	I have time for myself	0.62	0.97
V15-Tengo personas con las que puedo contar cuando las cosas salen mal	I have people I can count on when things go wrong	0.91	0.96
V16-Tengo a alguien que me cuida y se preocupa por mí	I have someone who takes care of and concerns about me	0.81	0.96
V17-Tengo a alguien con quien hablar sobre cómo me estoy sintiendo	I have someone to talk to about how I am feeling	0.88	0.96
V18-Si necesito orientación, hay alguien que me ayudará a elaborar un plan para enfrentar la situación	If I need advice there is someone who will assist me to work out a plan for dealing with the situation	0.89	0.96
V19-Tengo personas con las que puedo hablar y compartir mis experiencias	I have people I can talk to and share my experiences with	0.88	0.96
V20-Tengo personas que me mostrarán aprecio por la atención que le doy a mi bebé	I have people who will show me appreciation for the care I give to my baby	0.92	0.96
V21-Las personas cercanas a mí entienden que está bien que necesite ayuda	People close to me understand that it is okay for me to need help	0.89	0.96
V22-Puedo obtener comentarios positivos de los profesionales de la salud sobre mi capacidad para cuidar a mi bebé	I can get positive feedback from health care professionals about my ability to care for my baby	0.78	0.96
Factor 2. Apoyo práctico (apoyo informativo e instrumental)	Factor 2. Practical Support (informational and instrumental support)		
V01-Puedo obtener información sobre cómo alimentar al bebé	I can get information on how to feed the baby	0.92	0.96
V02-Puedo obtener información sobre cómo cambiarle el pañal / vestirlo	I can get information on how to change the nappies / dress the baby	0.93	0.96
V03-Puedo obtener información sobre cómo consolarlo / ponerlo cómodo	I can get information on how to console the baby / make the baby comfortable	0.92	0.96
V04-Puedo obtener información sobre cómo bañarlo	I can get information on how to bathe the baby	0.91	0.96
V05-Puedo obtener información sobre cómo cuidar mi cuerpo después del nacimiento del bebé	I can get information on taking care of my body after child birth	0.81	0.97
V08-Puedo obtener ayuda con mi bebé para alimentarlo	I can get hands on help with my baby to feed the baby	0.83	0.97
V09-Puedo obtener ayuda con mi bebé para cambiarle el pañal / vestirlo	I can get hands on help with my baby to change the nappies / dress the baby	0.92	0.96
V010-Puedo obtener ayuda con mi bebé para consolarlo / ponerlo cómodo	I can get hands on help with my baby to console the baby / make the baby comfortable	0.94	0.96
V011-Puedo obtener ayuda con mi bebé para bañarlo	I can get hands on help with my baby to bathe the baby	0.87	0.97

## Discussion

This study conducted for the first time the process of translation and validation of the original version of the functional social support domain of Patricia Leahy-Warren’s PICSS from English into Spanish.

Regarding face validity, the Spanish version of the functional social support domain had high comprehension, high clarity, and high precision. These coincide with prior studies in first-time mothers(10) and in experts,(11) who evaluated this domain as clear.

The Spanish version of the functional social support domain obtained high scores in the content validity index with respect to pertinence and relevance, evaluated by important experts from Colombia, which agrees with the high content validity presented in Ireland in prior studies.(7,11) With respect to construct validity, two factors were found, as reported by the PICSS author,(11) which also reflect conceptually the supporting presence (emotional and appraisal support) and the practical support (informational and instrumental support), evidencing that the Spanish version of this domain measures adequately the construct of functional social support in first-time mothers. 

The results of the distribution of the items in two factors obtained in this study agree with findings recently reported from Ireland,(11) except for the item “I can get information regarding baby care” that was placed in a different factor. Moreover, the study cited eliminated items; rather, in this study there was no need to eliminate any because the loads of the 22 items were > 0.3. The differences mentioned may be consequence of the adaptation made to the functional social support domain in another cultural context. 

In relation to the period of time in the postpartum in which the PICSS functional social support domain was applied and validated, the study conducted in Ireland(11) was applied in mothers at six weeks postpartum, while this study had participation of mothers up to six months postpartum, also finding the same two factors that represented 76% of the explained variance; hence, the present study broadens the time of the use of the domain in the postpartum, as a contribution to a valid and reliable measurement of functional social support in first-time mothers.

With respect to reliability, the high Cronbach’s alpha value presented by the Spanish version of the functional social support domain, for its 22 items and by factors, shows the shows the robustness of the validated domain. This agrees with the high Cronbach’s alpha values of the domain, reported in previous studies.(7,11) A limitation of this study is that it only included healthy mothers with term children, which is why future studies should include mothers with other conditions. In addition, it is recommended for each country to carry out the corresponding cultural adaptation of the functional social support domain.

The functional social support domain is a valuable tool in Spanish that only requires approximately 10 min for its self-completion, may be used during the hospital stay, upon discharge, and during postpartum follow up. In conclusion, given the robust psychometric properties of the Spanish version of the functional social support domain, health professionals can identify easily first-time mothers with low functional social support, conduct interventions that favor it, and evaluate the effectiveness of said health interventions, which is an important contribution to the nursing discipline in the area of maternal-infant health.
